# Androgen Receptor CAG Repeat Polymorphism and Epigenetic Influence among the South Indian Women with Polycystic Ovary Syndrome

**DOI:** 10.1371/journal.pone.0012401

**Published:** 2010-08-26

**Authors:** Shilpi Dasgupta, Pisapati V. S. Sirisha, Kudugunti Neelaveni, Kathragadda Anuradha, Alla G. Reddy, Kumarasamy Thangaraj, B. Mohan Reddy

**Affiliations:** 1 Molecular Anthropology Group, Biological Anthropology Unit, Indian Statistical Institute, Habsiguda, Hyderabad, India; 2 Department of Endocrinology, Osmania General Hospital, Hyderabad, India; 3 Anu Test Tube Baby Centre, Somajiguda, Hyderabad, India; 4 Centre for Cellular and Molecular Biology, Hyderabad, India; Universite de Montreal, Canada

## Abstract

The present study was carried out to assess the role of androgen receptor CAG repeat polymorphism and X chromosome inactivation (XCI) pattern among Indian PCOS women and controls which has not been hitherto explored and also to test the hypothesis that shorter CAG alleles would be preferentially activated in PCOS. CAG repeat polymorphism and X chromosome methylation patterns were compared between PCOS and non-PCOS women. 250 PCOS women and 299 controls were included for this study. Androgen receptor CAG repeat sizes, XCI percentages, and clinical and biochemical parameters were measured. The mean CAG repeat number is similar between the cases (18.74±0.13) and controls (18.73±0.12). The obese PCOS women were significantly more frequent in the <18 and >20 CAG repeat category than the lean PCOS women, yielding a highly significant odds (p = 0.001). Among the women with non-random X-inactivation, alleles with <19 repeats were more frequently activated among cases than controls (p = 0.33). CAG repeat polymorphism by itself cannot be considered as a useful marker for discriminating PCOS. We observed a trend of preferential activation of the shorter allele among the PCOS cases with non random XCI pattern. In the obese PCOS women, this microsatellite variation may account for the hyperandrogenicity to a larger extent than the lean PCOS women.

## Introduction

Polycystic Ovary Syndrome (PCOS), the leading cause of anovulatory infertility among premenopausal women, is now essentially known as androgen excess disorder [1]. It is characterized by three key features viz., hyperandrogenism, chronic anovulation or infrequent ovulation and presence of numerous follicular cysts in the enlarged ovaries. One of the mechanisms leading to the spectrum of pathological conditions deals with the abnormal steroid synthesis from ovaries and adrenals resulting in a hyperandrogenic state. Consequently, an excess of androgens seems to interfere with the process of follicular maturation leading to the PCOS phenotype. At the molecular level, the effect of androgens is mediated through the activation of androgen receptor (AR). The activity of AR is modulated by a polyglutamine tract of variable size in its N-terminal transactivation domain. This polyglutamine tract is encoded by a highly polymorphic CAG repeat sequence in exon 1 of the AR gene located on the X-chromosome [2].

In vitro studies showed an inverse relation between number of CAG repeats in the AR gene and AR activity [2], and a number of clinical conditions have been attributed to the resulting variation in androgen activity as listed by Mifsud et al. [3]. Thus, variation in the length of the AR (CAG)n repeats may provide insights into the underlying genetic etiology and further development of PCOS. In view of this, a number of studies have tried to investigate the role of CAG repeats of the AR gene in PCOS yielding contrasting results. Nevertheless, some of these studies reported a trend for shorter CAG alleles to be more frequent among PCOS cases than the controls [4], [5] which is consistent with the in vitro evidence of the greater receptor activity of the shorter CAG alleles. Apart from the microsatellite CAG repeat analysis, some of the studies have also looked into the X-chromosome inactivation (XCI) patterns among the cases and controls [4], [6]. However, these studies are largely restricted to the Caucasian populations. Neither the pattern of association of CAG repeat polymorphism with the PCOS nor the XCI patterns vis-à-vis the CAG repeat number are studied in any detail among the South-Asian women in general and particularly from India.

It is well known that the process of rapid urbanization and consequent lifestyle changes in India and other developing countries is increasing the burden of many complex diseases like PCOS, cardiovascular diseases and type-2 diabetes at an alarming rate and it is imperative to explore the nature of genetic and environmental etiology in the manifestation of such disorders among them. We have, in a relatively large cohort of South Indian women comprising of PCOS cases and healthy controls, investigated the role of CAG repeat polymorphism in the manifestation of PCOS and also studied the XCI pattern among them with the underlying hypothesis that shorter CAG repeat alleles would be preferentially activated in the women with PCOS.

## Results

### CAG allele distribution

**Figure 1:**
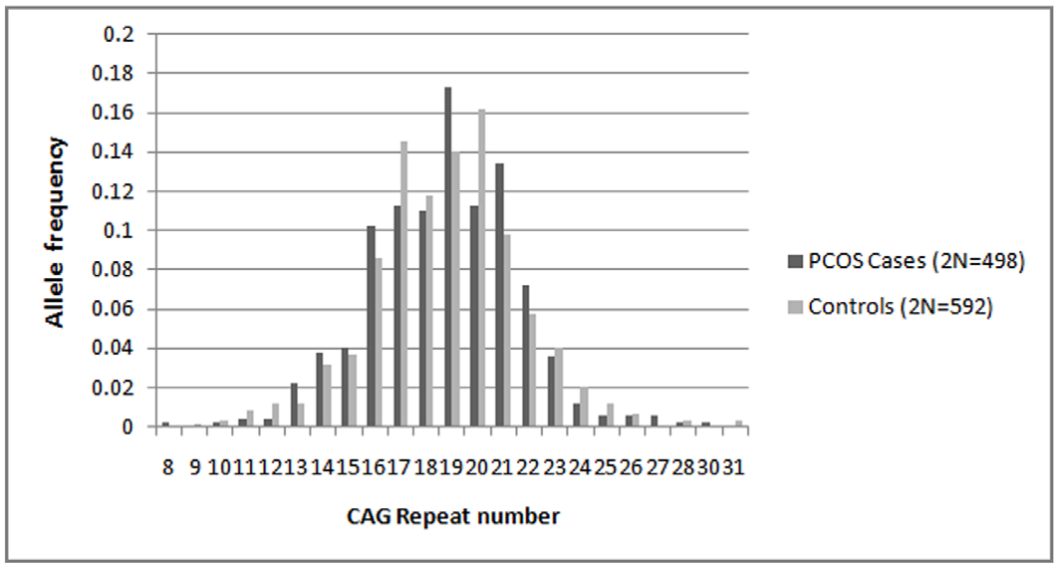
Distribution of CAG alleles in PCOS cases and controls.

In our cohort of 250 PCOS women and 299 controls, the androgen receptor CAG repeats ranged from 8–30 and 9–31, respectively (Fig.1). The mean CAG repeat number is almost identical between the cases (18.74±0.13) and controls (18.73±0.12), while the median value is same (19 repeats) for both. The allele distribution pattern was similar and not significantly different between cases and the controls {χ^2^ = 29.8, degrees of freedom (df) = 22, p = 0.122} (Fig.1). Both the cases and controls had a greater frequency of the biallelic mean CAG repeat sizes in the polymorphic range below the median value of 19 repeats. The frequency distribution of the PCOS and control women bearing repeat values below and above the median are presented in Table 1 and 2, separately for the total alleles (2N), biallelic mean, X-weighted biallelic mean and for both longer and shorter alleles. Contingency χ^2^ suggests homogeneity of distribution of cases and controls in the three qualitative categories of the repeat values (df = 2, p≥0.33), for each of the above allele categories.

**Table 1:** Distribution (%) of PCOS cases and controls according to the qualitative categories of CAG repeats.

	Total number of CAG alleles		CAG Biallelic Mean		CAG X-weighted Biallelic Mean	
Repeat size	Cases (2N = 498)	Controls (2N = 592)	Cases (N = 249)	Controls (N = 296)	Cases (N = 249)	Controls (N = 296)
<19	43.7	45.6	47.7	47.9	38.9	41.5
19	17.2	14	9.2	9.4	14.4	14.8
>19	38.9	40.3	42.9	42.5	46.5	43.5

χ2 = 2.18, df = 2, p = 0.336; χ2 = 0.212, df = 2, p = 0.899; χ2 = 0.513, df = 2, p = 0.773.

**Table 2:** Distribution (%) of PCOS cases and controls according to the qualitative categories of shorter and longer CAG repeats alleles.

	(i) Shorter CAG alleles			(ii) Longer CAG alleles	
Repeat size	Cases (N = 249)	Controls (N = 296)	Repeat size	Cases (N = 249)	Controls (N = 296)
<17	35.7	34.1	<20	38.5	37.1
17	18.0	16.5	20	14.4	18.5
>17	46.2	49.3	>20	46.9	44.2

χ2 = 0.561, df = 2, p = 0.755; χ2 = 1.668, df = 2, p = 0.434.

### Internal consistency in the CAG distribution patterns

Despite socioeconomic hierarchy and religious heterogeneity, the populations of Andhra Pradesh were found to be genetically homogenous [7]. Given that our sample consisted of sizeable cohort of Muslim subjects, we repeated the above analysis for the Hindu caste and Muslim subjects separately. But this categorization does not seem to have any significant effect on the allele distribution profiles (Table 3). Given that the Muslim and Hindu cases were predominantly drawn from the Osmania General Hospital and Anu Test Tube Baby Centre, respectively, and they in turn also represent lower and higher socioeconomic status, the results do not seem to suggest any effect of socioeconomic hierarchy in the pattern of manifestation of PCOS in relation to CAG repeats. Further, we drew 50, 60 & 70% random subset of case and control samples and repeated the above analysis to test for internal consistency in our data. Overall, the results suggest that the distribution of CAG repeats is homogenous (results not presented) between cases and controls in the entire polymorphic range indicating internal consistency.

**Table 3:** Distribution (%) of PCOS cases and controls according to the qualitative categories of CAG repeats in Hindus and Muslims.

	(i)Hindus		(ii)Muslims		(iii)Hindu Vs Muslim	
Repeat size	Cases (N = 158)	Controls (N = 218)	Cases (N = 81)	Controls (N = 61)	Hindu Cases (N = 158)	Muslim Cases (N = 81)
<19	48.1	47.2	48.1	52.4	48.1	48.1
19	7.6	9.6	12.3	11.4	7.6	12.3
>19	44.3	43.1	39.5	36.1	44.3	39.5

χ2 = 0.477, df = 2, p = 0.78; χ2 = 0.26, df = 2, p = 0.88; χ2 = 1.6, df = 2, p = 0.44.

Logistic regression was performed on the entire cohort of cases and controls to assess the association of repeat length (biallelic mean) with PCOS taking age and BMI as covariates, but the result was rather non-significant (OR = 0.993, p = 0.94). To examine if there is any effect of repeat numbers in the extreme ends of the allele spectrum on PCOS, odds ratio was also computed for two groups, (i) ≤15 repeat size VS >15 and (ii) ≤22 repeat size VS>22. Neither of the two analysis yielded any significant odds {(i) OR = 1.42, p = 0.581 and (ii) OR = 1.53, p = 0.366}. Further, even after merging the extremes of the two groups (i.e ≤15 and >22), no significant odds could be obtained (OR = 1.33, p = 0.49).

This is a situation in which we are unable to reject null hypotheses H_0_: θ = 0 {where, θ = log(odds ratio)} against H_1_: θ ≠ 0 (two-tailed alternative). We tried to estimate if our study with given sample size is sufficiently powered to reject null hypothesis, based on a previous study showing stronger association of shorter CAG alleles with PCOS with an estimated effect size of 0.318 in log of odds ratio [4]. Using G*Power software (version 3.1.0, Germany) [8], and given this magnitude of effect size, our sample has over 99% power to detect such an effect. We also estimated the minimum effect size, with which our study would have detected significant association (p = 0.05) with 90% power, which turns out to be 0.14. However, the effect size calculated from our data for CAG biallelic mean is 0.01, which is close to zero and far smaller than the minimum effect size estimated above to be able to reject null hypothesis with sufficient power. Conversely, our study can be considered as sufficiently powered in not rejecting the null hypothesis.

**Figure 2:**
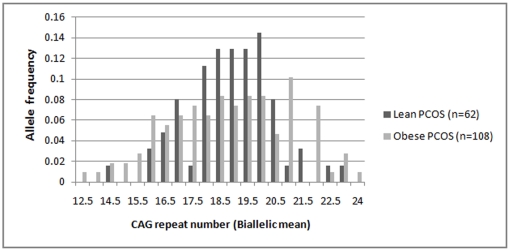
Distribution of CAG biallelic mean among Lean and Obese PCOS cases.

**Figure 3:**
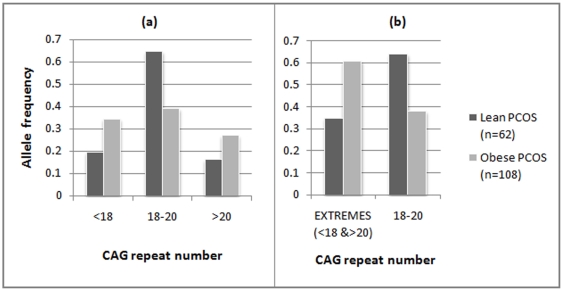
Qualitative distribution of CAG biallelic mean among Lean and Obese PCOS cases. (**a**) for the three categories,i.e, <18, 18–20 and >20, (**b**) for extreme CAG categories (<18 and >20) vs the middle range (18–20).

**Figure 4:**
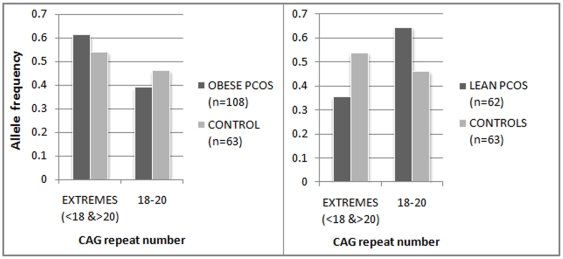
Relative frequency of extreme and middle range of CAG repeats among the obese PCOS/lean PCOS and controls.

The case cohort was also analyzed in two groups, based on body mass index (BMI), i.e. lean PCOS (BMI<25) and obese PCOS (BMI≥25) cases (Fig.2). Given the lean body mass of Indian women, we considered 25 to be the cut-off for distinguishing the lean women from overweight/obese women albeit as per the World Health Organisation (WHO) criteria, BMI >25 is considered overweight while BMI >30 is considered obese. While there was no significant difference in the allele distribution pattern when analyzed as separate cohorts, vis-à-vis the controls, we found significant difference in the biallelic mean distribution profile between the lean and obese PCOS cases (Fig.3a). Lean PCOS cases had significantly higher frequency (64.5%) of biallelic mean in the range of 18–20 than the obese cases (38.8%) (χ^2^  = 10.37, df = 2, p = 0.005). Conversely, the obese PCOS cases are found to be more frequent on either side of the middle range of the CAG repeats (<18 and >20). The significant difference between the lean and obese PCOS cases becomes much more pronounced when we consider both the extreme ranges of CAG repeats merged together in contrast to the middle range (χ^2^  = 10.37, df = 1, p = 0.001) (Fig.3b). This is pertinent given the plausible effect of CAG repeats in both the extremes in the androgen production and metabolism which could finally lead to a state of hyperandrogenism in a PCOS phenotype as demonstrated in previous studies [4], [6]. However, in comparison to controls, a greater proportion of the obese PCOS cases are again found in the CAG repeat range of <18 and >20, though statistically non significant (Fig.4). Similar analysis did not reveal any stratification in the CAG repeat number among the controls in function of BMI (p = 0.364).

Given significant heterogeneity in the pattern of CAG biallelic mean distribution of the obese and lean PCOS cases, odds were also computed for the pooled range of CAG repeats on both sides (<18 or >20) vis-à-vis the middle range (18–20), considering the pooled case cohort versus controls (Table 4) and the lean versus obese PCOS cases (Table 5). While in the former case, significant odds were not obtained at 5% level even after removing the effect of age and BMI, a highly significant odds were obtained (p = 0.001) in the latter (after removing age effect) to suggest that PCOS cases with repeat ranges <18 and >20, had 3 times greater risk to develop obesity and subsequent metabolic complications associated with PCOS. This particular regression analysis yielding highly significant odds ratio among the lean and obese PCOS women (Table 5) was further considered for post-hoc power analysis, specifying the odds ratio as 3.04, p-value as 0.05 and sample size as 170 (total number of lean and obese PCOS cases) as input parameters and with the conditional probability (under null hypothesis) of PCOS subject being obese taken as 0.63 for the patients with CAG repeat length of <18 or >20. This analysis yielded high power (1-β error probability) of 0.928, suggesting that our inference based on logistic regression has sufficient statistical power.

**Table 4:** Odds ratio for the CAG allele categories (<18 or >20) VS (18–20) in the entire case cohort versus controls taking age and BMI as covariates.

CAG Allele category	Covariates	B	S.E.	χ^2^	Df	p-value	OR	95% C.I.	
								Lower	Upper
	BMI	−0.24	0.03	60.9	1	0.00	0.78	0.73	0.83
	Age	0.06	0.02	5.8	1	0.02	1.06	1.01	1.11
(<18 or >20) vs (18–20)		**0.39**	**0.23**	**2.9**	**1**	**0.08**	**1.48**	**0.95**	**2.34**

**Table 5:** Odds ratio for the CAG allele categories (<18 or >20) VS (18–20) in the case cohort (for lean versus obese PCOS cases) taking age as covariate.

CAG Allele category	Covariates	B	S.E.	χ^2^	Df	p-value	OR	95% C.I.	
								Lower	Upper
	Age	0.06	0.04	2.83	1	0.092	1.06	0.98	1.14
(<18 or >20) vs (18–20)		**1.11**	**0.34**	**10.82**	**1**	**0.001**	**3.04**	**1.56**	**5.88**

### X chromosome inactivation (XCI) analysis

**Figure 5:**
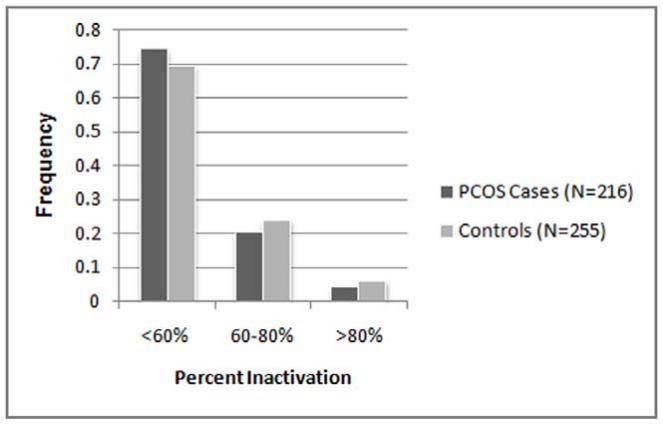
X-inactivation patterns in PCOS cases and controls.

**Figure 6:**
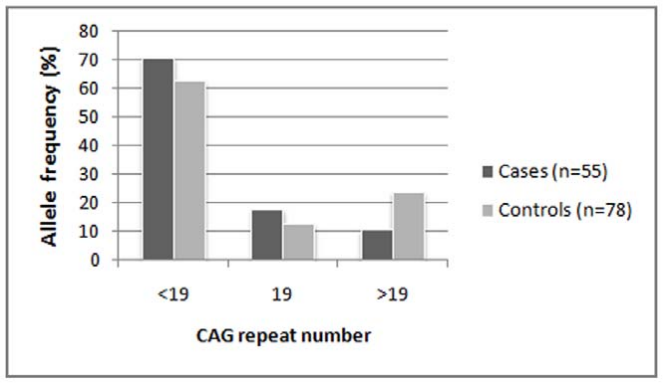
Distribution of CAG alleles with 60–100% activity among women with non-random X-Inactivation pattern.

Analysis for X-chromosome inactivation revealed that majority of the cohort (74% of the PCOS cases and 69% of the controls) follow random (<60%) X-inactivation pattern with reference to the CAG repeats (Fig.5). Nonrandom X-inactivation (60%–80%) was observed only in 21% of the PCOS cases and 24% controls. This difference in XCI pattern between cases and controls is neither significant nor follow the expected trend of cases showing relatively greater proportion of nonrandom inactivation. However, among the subset of samples with non-random X-inactivation, alleles with <19 repeats were preferentially more activated among the PCOS women (71%) as compared to the controls (63%), though the difference was not statistically significant (p = 0.33) (Fig.6). Logistic regression analysis for preferential activation of shorter alleles yielded an odds ratio of 1.443 though not significant (p = 0.33). With reference to the effect size {log(odds ratio) = log(2) = 0.318} obtained in the previous study [4], our sample of 133 individuals with non-randon X-Inactivation gives a power of 95.6%. With our sample size, the expected minimum effect size to achieve significant association at 90% power is estimated to be 0.281.

X_weighted_biallelic mean was calculated by incorporating XCI percentages and their distribution was similar to that of the biallelic mean distribution (Table 1), which did not show any difference between cases and controls, hence no perceptible epigenetic influence. This analysis was also done separately for Hindus and Muslims and for lean and obese PCOS cases, but the results exhibited essentially the same trend of X-inactivation. Logistic regression was also performed on the entire cohort of cases and controls to assess the association of X-weighted biallelic mean with PCOS taking age and BMI as covariates. The odds was non-significant (OR = 1.11, p = 0.54). For the post-hoc power analysis, the expected effect size is estimated to be 0.24 from the earlier data [4], for which the power obtained with our sample was close to 1 (0.99).

### Clinical correlation

We obtained the testosterone (T) levels and hirsutism score of the PCOS cases (Table 6) and found that the frequency of PCOS women was not significantly different across the allele categories with respect to T levels in the pooled sample. Independent analysis of Hindu and Muslim samples resulted in the same pattern (results not presented). However, 72% of the obese PCOS cases with repeat length ≤ 19 show high testosterone values as compared to the lean PCOS with same range of CAG repeats (31%), although this difference in the proportions do not reach statistical significance (p = 0.15). On the other hand, the clinical measure of hyperandrogenism as reflected by hirsutism score, was found to be significantly more frequent among the obese when compared to the lean PCOS (χ^2^ = 9.9, df = 1, p = 0.002), in concurrence to the highly significant correlation observed between hirsutism and BMI (r = 0.403, p<0.001). However, testosterone levels and hirsutism were not significantly correlated with either biallelic mean or with X_wt_biallelic mean in the pooled cohort of case.

**Table 6:** Androgenic characteristics among PCOS cases under different biallelic mean categories.

	TESTOSTERONE						HIRSUTISM (FG SCORE)		
	Low T Group (<0.67 ng/dl)			High T Group (>0.67 ng/dl)					
Biallelic Mean	N	Range	Mean±SE	N	Range	Mean±SE	N	Range	Mean±SE
<19	39	0.01–0.67	0.43±0.02	45	0.7–27.9	2.15±0.64	107	0–14	5.5±0.37
19	3	0.36–0.61	0.52±0.08	10	0.68–1.4	0.93±0.07	22	0–18	4.4±0.88
>19	37	0.07–0.67	0.42±0.02	40	0.68–33.3	2.17±0.82	90	0–20	6.15±0.41
Total	79		0.43±0.02	95		2.03±0.46	219		5.68±0.27

## Discussion

Given the variation in the CAG repeat length of the androgen receptor (AR) gene and its inverse effect on the receptor activity, the alleles with short CAG repeat length are expected to result in amplified androgen receptor activity, resulting in a state of hyperandrogenism. Thus, we hypothesized that the shorter alleles would be more frequent and preferentially more active among the PCOS women than the controls. To the best of our knowledge, this is the first study of its kind on a large cohort of Indian women to examine the association of AR polymorphism with PCOS. Our study revealed a range of 8–31 CAG repeats in the AR gene of South Indian PCOS women and the controls. However, neither the distribution of biallelic mean values nor the mean repeat sizes were found significantly different between the PCOS cases and controls. These results were concurrent to the observations of some previous studies which did not find significant difference in the mean values of CAG repeat sizes between PCOS cases and controls [3], [9]–[14]. However, there have been other studies which yielded divergent and contradictory results; while Xita et al [5] and Shah et al [4] found shorter alleles to be more frequent among the PCOS cases than controls, Hickey et al [6] found longer alleles to be significantly higher in frequency in the PCOS women than controls. This inconsistent nature of association could be in part accounted for by the variation in the CAG repeat number in different populations and ethnic groups as observed by Edwards et al. [15]. Our study did not reveal any association of CAG alleles, shorter or longer, with PCOS; the distribution of the cases and controls appear to be highly homogeneous across the spectrum of CAG alleles, with odds ratio close to 1 and effect size zero. Based on expected effects as presented in a previous study and given our sample size the minimum effect size expected would 0.14 to be able to detect significant association with power of 90%.Therefore, our study can be regarded as sufficiently powered to replicate the previous association between CAG repeats and PCOS.

Since AR gene is located on X chromosome, the epigenetic phenomenon of X chromosome inactivation leading to preferential activation of either the shorter or the longer allele may explain the differential androgen receptor activity resulting in the phenotypic or clinical heterogeneity in PCOS, chiefly the androgenic features. XCI analysis has been previously done in both PCOS and non-PCOS cases with hyperandrogenic features. Calvo et al. [16] found no significant difference in the pattern of X inactivation between women with hyperandrogenic hirsutism/idiopathic hirsutism and controls. Subsequently, Shah et al [4] demonstrated preferential inactivation of the longer CAG repeat alleles resulting in expression of the smaller alleles that predictably increase the androgen sensitivity. However, XCI analysis revealed random pattern of X-inactivation of the relatively larger proportion of our Indian samples and sub-samples. Nevertheless, concurrent to the functional hypothesis, analysis of samples with non-random XCI revealed that shorter alleles were preferentially more activated among the PCOS cases than controls, although the relative proportion of the activated alleles were not significantly different between them (OR = 1.443, p = 0.33). This trend is consistent with Shah et al [4] observation which suggests that increased intrinsic androgenic activity associated with short AR alleles may have a role in the pathogenesis of PCOS-related hyperandrogenism.

Hyperandrogenism, which is ascertained biochemically by assessing the testosterone levels, and clinically by hirsutism using the Ferriman-Gallwey score, was also taken as a parameter for analyzing its association with CAG repeat numbers. We did not observe any significant association between testosterone levels and CAG repeat length which is in contrast to some of the previous studies [3], [5], [6], [13], [17]. Consistent with the functional hypothesis, although there is a trend of shorter CAG alleles showing lower levels of testosterone than those with longer CAG alleles in the PCOS women, it is not statistically significant unlike in case of the previous studies [3], [6], [13]. Given the relatively large sample size, this trend cannot be therefore considered as suggesting a relatively greater role of excessive androgen action (implying receptor activity) in PCOS than expected from hyperandrogenemia alone. The obese PCOS cases of our study exhibited contrasting trend in comparison to the lean cases, in that the shorter CAG repeat length is associated with a high testosterone value, albeit statistically not significant, supporting the hypothesis that increased androgen activity in women has a stimulatory effect on ovarian androgen production [17].

The pattern of body fat distribution can regulate androgen production and metabolism to a significant extent [18]. Both production rates and metabolic clearance rates of androgens are equally increased in obesity. A condition of relative hyperandrogenism in women are associated with abdominal obesity and all the features of the metabolic syndrome, including insulin resistance and compensatory hyperinsulinemia, low high-density lipoprotein(HDL) cholesterol, increased triglycerides, and elevated arterial blood pressure [18] that are commonly present in a PCOS phenotype. Thus, we also examined the association of hyperandrogenic features in obese and lean PCOS cases separately vis-à-vis the CAG repeat numbers. When CAG repeat distribution analysis was carried out among the lean and obese PCOS cases, there was a significant difference between them (Fig.3a) whereby the lean PCOS cases had significantly higher frequency of biallelic mean in the middle range of 18–20 than the obese cases. Subsequent logistic regression analysis yielded significant odds ratio with a high statistical power of 92.8% suggesting that the PCOS women with CAG biallelic mean range of <18 and >20 are at a much higher risk to develop obesity and associated metabolic complications. However, there seemed to be no epigenetic influence over this heterogeneity, since majority of the cases followed random XCI pattern. Apart from this, we also found a significant positive correlation (r = 0.403, p<0.001) between hirsutism and BMI within the PCOS cases. Among the hirsute cases, the proportion of obese PCOS women is significantly (p = 0.02) higher (77%) than the lean PCOS women (23%). This could be explained by the increase in the metabolic clearance rate of androgen, particularly in obese women which finally leads to augmented hyperandrogenic features like hirsutism.

To sum up, we found homogeneity in CAG allele distribution profile of the cases and controls, hence CAG repeat polymorphism, by itself, cannot be considered as a useful discriminator between the PCOS cases and controls. Further, in spite of the lack of evidence for a major epigenetic effect, we observed a trend of preferential activation of the shorter allele among the PCOS cases with non random XCI pattern. On the other hand, the significant heterogeneity in the CAG biallelic mean between the obese and lean PCOS cases can explain the differential receptor activity among them, which may also probably explain the manifestation of hyperandrogenic features in such cases. Thus, the lean PCOS women, who have a significantly higher frequency of the middle range CAG repeats, would possibly be conferred with a moderate receptor activity as compared to the obese PCOS cases. In the latter group, the lower or higher CAG repeat numbers of the polymorphic spectrum would therefore lead to an increased or diminished receptor activity, respectively, both of which are expected to result in a hyperandrogenic state. We may further add that in case of the lean PCOS group the androgen receptor CAG repeats may not play a very instrumental role in manifestation of the hyperandrogenic features, while in the obese PCOS group this microsatellite variation may account for the hyperandrogenicity to a larger extent. XCI analysis among both these groups also revealed the same pattern as observed in the entire cohort implying that the epigenetic effect may not have any major effect on the androgen metabolism and receptor activity among Indian PCOS women. Overall, our results rule out the possibility of suggesting CAG repeat polymorphism as a prognostic marker for PCOS. Especially given that ours is one of the largest samples so far studied and from a relatively more homogenous population and due to internal consistency observed in the sub samples within our sample, we may repose fair degree of confidence in the reliability of our results. Nonetheless, our study is the first of its kind from this region and therefore further replicative studies are required among other ethnic groups, encompassing the geographic heterogeneity of India before reaching any unequivocal conclusions on the precise role of androgen receptor CAG repeat polymorphism in the manifestation of this immensely heterogeneous PCOS phenotype in the populations of this region.

## Materials and Methods

### Study Population

As part of the larger project “Identification of susceptibility genes for Polycystic Ovary Syndrome among South-Indian women”, undertaken by us at the Indian Statistical Institute, a total of 549 women consisting of 250 PCOS cases (aged 14–40 years) and 299 controls (aged 14–47 years) were recruited for the study. Patients were enrolled from the Gynecology clinic of the Osmania General Hospital, Hyderabad, as well as from an infertility clinic (Anu Test Tube Baby Centre, Hyderabad), as per the Rotterdam criteria, 2003 [19], which stipulates having any two of the following three conditions to qualify for the inclusion: (i) presence of clinical and/or biochemical signs of hyperandrogenism, (ii) infrequent periods with intermenstrual interval of more than 35 days, and (iii) polycystic ovaries; an ovary with the ultrasound appearance of more than 10 subcapsular follicles (<10 mm in diameter) in the presence of prominent ovarian stroma was considered polycystic. Patients with hyperprolactinemia, thyroid and adrenal diseases, 21-hydroxylase deficiency, and androgen-secreting tumours were excluded. The weight and height of the 170 PCOS cases and 238 control subjects were recorded. Hirsutism, defined as a Ferriman-Gallwey score of more than 5 [3], could be recorded for 219 of the 250 PCOS cases. Androgen parameters, testosterone (T) and DHEA-S, were recorded for 174 cases. Normal controls with no history of treatment for fertility, and with normal menstrual cycles every 25–32 days were recruited from the family planning centre of the Osmania hospital and from the general population. Intravenous blood samples (∼5 ml) were collected from both the patients and controls after obtaining their informed written consent. The study protocol was approved by the Indian Statistical Institute Review Committee for Protection of Research Risks to Humans.

### DNA extraction, amplification and Genescan analysis

DNA was extracted from the peripheral blood samples of the patients and control using the phenol-chloroform method [20]. The CAG repeats were genotyped using a PCR-based assay. Genomic DNA was amplified by PCR using fluorescently labeled primers that flank the CAG repeats [21]. The forward primer was labeled at the 5′end with the dye 6FAM. Amplification was carried out in a GeneAmp9700 thermal cycler (Applied Biosystems, Foster City, CA) as previously described [21]. The amplified products were separated on a denaturing polyacrylamide gel using an ABI 3730 genetic analyzer (Applied Biosystems, Foster City, CA). The fragment size was estimated by comparison with the internal size standard GS-LIZ500.

### X-Chromosome inactivation (XCI) analysis

X inactivation analysis was carried out using the protocol given by Hickey et al [6]. For the 471 heterozygous subjects, 100 ng of DNA were either digested with 1U of HpaII or incubated in digestion buffer alone at 37°C overnight, followed by incubation at 95°C for 5 min to denature the enzyme. Next, 1 µl of digested and mock-digested products were amplified using PCR primers given above and peak areas of both the alleles were determined using an ABI 3730 genetic analyzer and Genemapper (version 3.7, Applied Biosysytems). All samples were analysed in duplicate in digested and nondigested conditions. The accuracy of the results obtained through Genescan has also been verified through direct sequencing.

X inactivation (relative methylation of each allele) was quantified as previously described by Hickey et al. [6], comparing the ratio to which each allele contributed to the total peak area between digested and undigested samples. The X-Inactivation percentages are expressed in terms of degree of inactivation of the longer allele. Nonrandom X inactivation is defined as more than 60% inactivation of either allele; skewed X inactivation is defined as more than 80% inactivation of either allele.Following X-Inactivation analysis, we calculated X-weighted biallelic means as per the protocol given by Hickey et al. [6], whereby each allele in a genotypic pair is multiplied by its percent activation, and the two adjusted repeat values are added together.

### Statistical Analysis

All the statistical analyses were performed with the help of SPSS statistical software (version 15.0, SPSS Inc, Chicago, IL, USA) and Minitab (version 15). For power calculation, G*Power Software (version 3.1.0) was used.
